# Temporal and Spatial Influences on Fawn Summer Survival in Pronghorn Populations: Management Implications from Noninvasive Monitoring

**DOI:** 10.3390/ani14101468

**Published:** 2024-05-15

**Authors:** Cole A. Bleke, Eric M. Gese, Juan J. Villalba, Shane B. Roberts, Susannah S. French

**Affiliations:** 1 Department of Wildland Resources, Utah State University, Logan, UT 84322, USA; juan.villalba@usu.edu; 2 U.S. Department of Agriculture, Wildlife Services, National Wildlife Research Center, Utah Field Station, Logan, UT 84322, USA; eric.gese@usu.edu; 3 Idaho Department of Fish and Game, Boise, ID 83712, USA; shane.roberts@idfg.idaho.gov; 4 Department of Biology, Utah State University, Logan, UT 84322, USA; susannah.french@usu.edu

**Keywords:** forage resources, life-history stages, noninvasive sampling, nutrition, population management, pronghorn antelope

## Abstract

**Simple Summary:**

Monitoring vital rates allows managers to estimate trends in growth rates of ungulate populations, but connecting the influence of nutrition on ungulate demography is challenging. Internal and external factors are likely to influence neonate survival and recruitment. We found that nitrogen available to adult female pronghorn during early lactation had the greatest influence on fawn summer survival (recruitment). Pronghorn management, where fecal sampling is utilized, should be conducted at the subpopulation level and have baseline fecal nitrogen measures taken. Subpopulations with low recruitment can be positively influenced by increasing nitrogen, or protein, available to them during the early lactation period.

**Abstract:**

Monitoring vital rates allows managers to estimate trends in growth rates of ungulate populations. However, connecting the influence of nutrition on ungulate demography is challenging. Noninvasive sampling offers a low-cost, low-effort alternative for measuring nutritional indices, allowing for an increased understanding of the mechanistic relationships between environmental factors, nutrition, and specific population vital rates. We examined the temporal influence of intrinsic and extrinsic factors on pronghorn (*Antilocapra americana*) fawn recruitment. We collected fresh fecal samples from adult female pronghorn in five subpopulations spanning three sampling periods associated with critical maternal life-history stages (late gestation, early lactation, breeding season) for 2 years to investigate both intra- and interannual influences. Intrinsic factors were fecal glucocorticoid metabolites (FGMs), nutritional indices (fecal nitrogen (FN) and 2,6-diaminopimelic acid (DAPA)), and dietary composition (protein intake of forbs, graminoids, legumes, other, shrubs), while the extrinsic factor was vegetative greenness (normalized difference vegetation index (NDVI)). We found variations in DAPA, protein intake of forbs, variation in forb protein intake, and protein intake of legumes during late gestation positively influenced fawn recruitment. Fecal nitrogen during early lactation showed the strongest positive influence on the recruitment of any measured parameter. Finally, breeding season NDVI and the variation in DAPA values positively influenced the subsequent year’s fawn recruitment. Our longitudinal study enabled us to investigate which parameter was most important to specific periods of fawn development and recruitment. We combined the results across five subpopulations, but interpretation and subsequent management decisions should be made at the subpopulation level such that pronghorn subpopulations with low recruitment can be positively influenced by increasing nitrogen on the landscape available to adult females during the early lactation period. As the use of noninvasive monitoring methods continues to expand, we believe our methodologies and results can be broadly applied to other ungulate monitoring programs.

## 1. Introduction

Reliable estimates of vital rates, such as survival and reproduction, are required for both management and scientific understanding of ungulate population dynamics [1]. While there have been some attempts to formally predict population dynamics from harvested ungulates using statistical models [2,3,4], robust and widely applicable prediction tools are currently rare [5]. Monitoring population vital rates (i.e., pregnancy, litter size, neonatal and adult survival) allows managers to estimate trends in population growth rates, as well as mechanistic relationships between environmental factors and specific vital rates of population demography [6]. Juvenile survival in herbivores tends to exhibit greater spatial and temporal variation than adults and, as a result, can have a larger influence on population dynamics [7,8]. Therefore, predicting the future juvenile survival of large herbivores would greatly improve population dynamics modeling and management [5]. Age ratios (i.e., young:adult) are indices regularly incorporated into many wildlife monitoring programs and widely collected via herd composition surveys to infer demographic trends [9]. Ungulate age ratios are used to estimate fecundity and survival rates of young in harvested, threatened, and endangered populations [7,10,11,12,13]. Most commonly, age ratios index recruitment, which is the product of fecundity and survival of young [14]. Harris et al. [9] suggested age ratios can meaningfully reflect the dynamics of an individual component of the ratio, specifically the survival of the young (i.e., recruitment) in wapiti (*Cervus canadensis*).

Traditionally, physical capture has been the practice for obtaining population information from large mammals [15]. Capture–mark–recapture methodologies can provide accurate and unbiased estimates of population vital rates, such as survival, but are costly [16,17,18] in comparison to noninvasive methods [18]. Noninvasive methods for population monitoring are becoming important components in modern conservation practices, given they are relatively low-cost and low-effort sampling for estimation of individual and population statistics [19,20]. The ability to collect repetitive biological samples containing physiological information makes urine and fecal sampling superior to invasive methods (e.g., capture and handling), which may compromise the physiological state of wild animals [21] while presenting potential hazards to both the animal [22] and the handler [23]. Fecal sampling has many advantages for the biological matrices (e.g., blood, saliva, excreta, integumentary structures) used for measuring hormone metabolites. This method allows for a more accurate and integrated measure of the hormone levels in circulation, considering that metabolites are excreted over a period of hours [24,25] or up to a day, depending upon gut retention time in mammals [24,26,27]. In addition, feces can be easily collected, making temporally longitudinal studies possible [28].

Glucocorticoids are the primary stress steroid hormone in mammals and play a fundamental role in the regulation of energy homeostasis in relation to life-history events (e.g., growth, pregnancy, lactation, migration; [29,30,31]). Glucocorticoids are produced via plasma glucocorticoids, metabolized by the liver and other organs, excreted into the urine via the kidneys or into the gut via bile ducts, and expelled into fecal matter [5]. Fecal glucocorticoid metabolites (FGMs) reflect the free, or unbound, fraction of total glucocorticoids [32,33]. Researchers can use stress measurements (i.e., FGMs) obtained from fecal samples as a tool to evaluate the condition of an ungulate population during particular periods or in specific areas to monitor the relationship between animals and their environment [34], as well as the influence of human disturbances on animal species [35,36].

Knowledge of how nutrition influences the physiological condition of the pregnant female and the subsequent survival of her offspring is paramount to increasing our understanding of the mechanistic linkages between the environment, nutritional condition, and population performance of large ungulates. Nutritional status influences maternal body condition, pregnancy, body size, and survival of both the female and neonate [37], reflecting the quality of forage available to the female during gestation [38]. The quality of a diet is an essential driver of the performance of individuals, which in turn affects the dynamics of populations [39]. In turn, this variable can be used in models of population dynamics to better understand how habitat and nutritional conditions affect ungulate populations, a relationship that is necessary for informing management decisions, in particular for areas experiencing declining ungulate population trends [40,41]. Protein is often seasonally limited for wild ruminants, which has prompted research to focus on indices designed to estimate its concentration in animal diets [42]. As a result, multiple methods have been developed to monitor the diet quality of free-ranging ungulates. These methods are of growing relevance given the dynamic relationship between dietary composition and changes in climate [43,44]. The utilization of indirect measures of diet quality (i.e., fecal indices) is a valuable option based on both the feasibility of sample collection and the minimal disturbance, if any, caused to the animal [45]. Two commonly used fecal indices are fecal nitrogen (FN), an estimate of dietary nitrogen and diet quality [46,47], and fecal 2,6-diaminopimelic acid (DAPA), an estimate of rumen bacterial mass and an index of digestible energy [48,49,50,51].

Nutrition is impacted by changes in forage species availability, abundance, and plant phenological stage [41]; thus, animals must optimize their diet and forage intake to meet their dynamic nutrient demands [52]. Ruminants are categorized by feeding type, and those labeled as intermediate or mixed feeders, like pronghorn antelope (*Antilocapra americana*), are generally selective but opportunistic foragers [53]. This is often a function of small body size and relatively small rumen, which limits their ability to digest plants high in lignocellulose, such as graminoids, resulting in its avoidance when other functional groups are available [53]. However, there are positive associative effects from protein-rich forages (e.g., forbs) in diverse ruminant diets, which allow for more efficient utilization of plants high in fiber, like graminoids [54].

Pronghorn antelope are a small ruminant (45 kg) native to sagebrush (*Artemisia* spp.) and grassland ecosystems in western North America [38,55]. Pronghorn have the unique distinction among mammals to be the only members of the family Antilocapridae and genus *Antilocapra* [56]. The current distribution of pronghorn is but a fraction of their historic range [57], prompting research into techniques for monitoring population status. They are particularly sensitive to stress and mortality during physical capture [58], and myopathy is not an uncommon consequence of handling [12,59], making alternative methods such as noninvasive sampling appealing. The goal of this study was to determine when and what maternal measure had the largest influence on pronghorn fawn recruitment. Thus, we designed a population monitoring study utilizing noninvasive fecal sampling from free-ranging, adult female pronghorn where our objectives were to (1) examine intra-annual intrinsic and extrinsic factors influencing fawn recruitment, and (2) explore interannual influences (i.e., lag effects) of intrinsic and extrinsic factors on the following year’s recruitment. The intrinsic factors included adult female hormonal measures (e.g., FGM (ng/g)), nutrition indices (e.g., FN (%) and DAPA (mg/g)), and diet composition (e.g., protein intake of forbs, graminoids, legumes, other, shrubs (%)); while the extrinsic factor was vegetative greenness (i.e., normalized difference vegetation index (NDVI)) within each study site. We predicted that (1) there would be temporal differences in correlations between the various predictor variables and pronghorn summer fawn survival, based on seasonal metabolic demands of the female, (2) that adult female nutritional indices (i.e., FN and DAPA) and NDVI (a proxy for plant productivity) would positively influence fawn summer survival during each seasonal collection period, and (3) the nutritional condition of the doe during or at the beginning of the breeding season would positively influence the following year’s fawn summer survival.

## 2. Materials and Methods

### 2.1. Study Area

**Figure 1:**
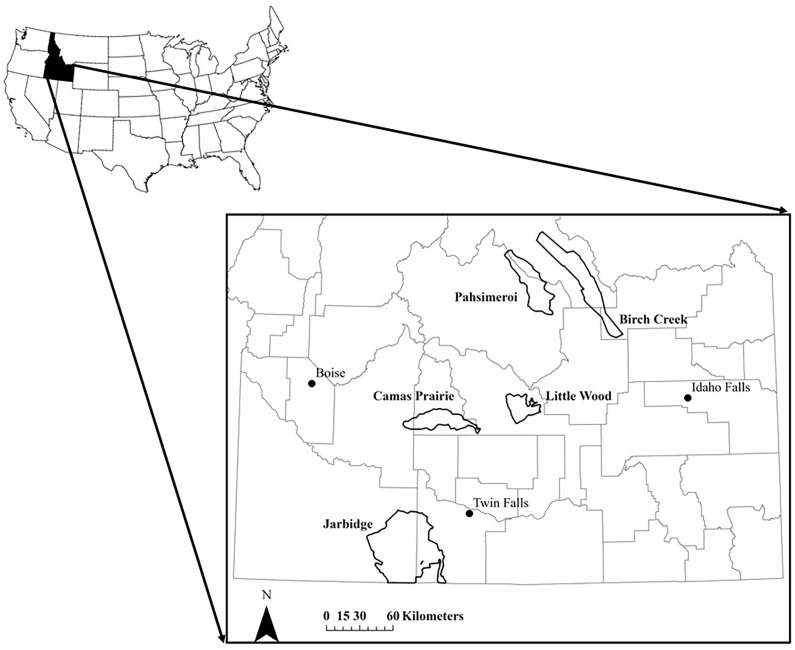
Pronghorn antelope summer distributions. Ranges of the five study subpopulations (Birch Creek, Camas Prairie, Jarbidge, Little Wood, Pahsimeroi) of pronghorn antelope within the state of Idaho. Grey lines represent county boundaries.

We studied five subpopulations representing the majority of pronghorn habitats and population productivities in southern Idaho, USA [47] (Figure 1), including Jarbidge, Camas Prairie, Little Wood, Birch Creek, and Pahsimeroi (Table 1). The Jarbidge site had a resident pronghorn subpopulation occupying a desert habitat. Based on Idaho’s gap analysis land cover classification [60], basin and Wyoming big sagebrush (*Artemisia tridentata tridentata* and *A. t. wyomingensis*) were the dominant cover types. Perennial grasslands (i.e., crested wheatgrass (*Agropyron cristatum*)) were the next dominant cover type, with the remaining landscape being a mix of low sagebrush (*Artemesia arbuscula*), antelope bitterbrush (*Purshia tridentata*), and rabbit brush (*Chrysothamnus* spp.) communities. The Camas Prairie site had a migratory pronghorn subpopulation persisting largely on agricultural lands through the summer months. Alfalfa (*Medicago sativa*) was the dominant crop with planted grassland parcels enrolled in the Conservation Reserve Program [61]. The Little Wood site had a migratory pronghorn subpopulation occupying native shrub–steppe rangelands with big sagebrush and irrigated agriculture throughout. Birch Creek and Pahsimeroi study sites contained migratory subpopulations inhabiting mountain valley habitats. The Birch Creek subpopulation occupied both the Birch Creek and Lemhi valleys, where low sagebrush was the dominant vegetation community. Mountain, basin, and Wyoming big sagebrush also accounted for a large portion of the study site with limited agricultural lands. The Pahsimeroi subpopulation occupied both the Pahsimeroi and Little Lost River valleys, where mixed stands of mountain big sagebrush and low sagebrush dominated the landscape. Basin and Wyoming big sagebrush were the next most abundant cover types, and agricultural lands were also limited [47]. See Bleke et al. [62] for additional descriptions of habitat types.

### 2.2. Sample Collections

We randomly collected fresh fecal samples from unmarked, reproductive-aged female pronghorn (i.e., ≥2 years) in 2018 and 2019. Collections occurred during three sampling periods selected to coincide with maternal life history stages: late gestation (April to mid-May), early lactation (June), and breeding season (September). We discuss our results in accordance with female life history stages because we were unable to associate pregnancy or lactation status with individual adult female pronghorn samples, given our noninvasive sampling design. We used magnifying optics to categorize individuals by age class (i.e., fawn, yearling, adult, unknown) and sex and to monitor defecation. Females were identified by a lack of a black cheek patch. We used 2-person teams with two-way radio communication to locate fresh pellet piles. If we were uncertain whether an individual female pronghorn was sexually mature (e.g., a lone individual that lacked a reference adult female for age or size comparison) or we believed an individual was a yearling based on estimated shoulder height or muzzle length, we did not collect a sample. We collected fecal samples from spatially segregated groups of animals to obtain a representative sample of the subpopulation and avoid duplicate sampling.

### 2.3. Laboratory Methodologies

We collected a total of 1440 samples during our study. From those, we randomly selected 20 samples/subpopulation/sampling period/year (*n* = 560 samples; 2018 = 260, 2019 = 300) for analyses of fecal glucocorticoid metabolites [63], diet composition via plant DNA barcoding [62], and nutritional indices via fecal nitrogen and DAPA [31]. The overall percentage component of dietary protein, for functional group or family- and genus-level from each operational taxonomic unit, was calculated by summing all samples and dividing by the total [64]. The Little Wood and Camas Prairie subpopulations were not sampled during late gestation in 2018. We dried fecal samples in a drying oven (Precision Scientific, Chicago, IL, USA), where temperatures were held below 50 °C until all moisture evaporated. We ground dried samples with a coffee grinder (Hamilton Beach, Southern Pines, NC, USA) until fecal material became a consistent powder in texture. We sent portions of the dried and ground samples to the Wildlife Habitat and Nutrition Laboratory at Washington State University (Pullman, WA, USA) to measure concentrations of DAPA and fecal nitrogen. DAPA was calculated following the methods of Davitt and Nelson [48], while fecal nitrogen percent was determined using a TruSpec CN Analyzer (LECO, St. Joseph, MI, USA). We also sent portions of the same samples to Jonah Ventures Laboratory (Boulder, CO, USA) for plant DNA barcoding analyses; the laboratory methodologies are summarized in [62]. We used cortisol enzyme-linked immunosorbent assay (ELISA, ADI-900-071, Enzo Life Sciences, Inc., Farmingdale, NY, USA) kits to measure FGM concentrations. We followed established protocols that were optimized for pronghorn [63] for fecal steroid metabolite extractions, validations, and measurements. Fecal glucocorticoid metabolite measurements were calculated in 34 assays with a mean intra-assay variation of 1.96% and an inter-assay variation of 15.42%.

### 2.4. Herd Composition Surveys

We conducted ground-based pronghorn composition surveys once per year for each subpopulation to estimate sex and age ratios (i.e., sex (male:female) and age (young:adult)) as a proxy for fawn summer survival. Surveys occurred between 30 July and 6 August 2018–2020. Pronghorn are most dispersed during the fawning season (i.e., May through June), but fawns are in their hiding stage, preventing accurate herd structure estimates [65]. Group sizes remain small and relatively dispersed during August [66] when fawns have increased their activity and grown out of the hiding stage, making late summer an ideal time to calculate pronghorn fawn summer survival [67]. We will henceforth refer to fawn summer survival as fawn recruitment.

We did not survey the Little Wood pronghorn in 2018 due to a wildfire. We began each survey at sunrise and completed each prior to 1200 h. We used one vehicle per survey with two observers, a driver and passenger, who were either Idaho Department of Fish and Game (IDFG) biologists or personnel experienced with pronghorn sex and age classifications. Vehicles maintained speeds of ≤40 km/h and stopped every 0.80 km along the route. Observers visually panned their respective 180° perpendicular to the vehicle using magnifying optics (i.e., binoculars and spotting scopes) at each stop and recorded all pronghorn observed. Observers recorded the location of the vehicle with a handheld GPS (Garmin Ltd., Olathe, KS, USA) and the composition of the group (i.e., adult male, adult female, male or female yearling, young, unknown). We attempted to delineate nonreproductive-aged (i.e., yearling) from reproductive-aged (i.e., ≥2 years) females during surveys to increase precision in our recruitment estimates [65]. Several years of these same surveys in various subpopulations of pronghorn were used to examine the influence of landscape variables and climate on fawn recruitment across Idaho [68].

### 2.5. NDVI

We used atmospherically corrected Landsat 8 surface reflectance data from the Google Earth Engine [69,70] to calculate average NDVI values at 30 × 30 m resolution for each subpopulation’s summer range. We defined these summer ranges with the assistance of IDFG biologists based on their knowledge of these areas. We collected mean NDVI values of each summer range during the three sampling periods, which represented the temporal variation, as well as the standard deviation of the summer ranges, which represented the spatial variation.

This index is the normalized reflectance difference between the visible red bands and the near-infrared, which tracks chlorophyll quantity and plant production throughout the growing season [71,72,73]. We interpolated missing NDVI values across time using the “interpolation” command in the impute time series library (imputeTS; [74]). We standardized the NDVI values to a −1 to 1 scale to facilitate comparisons. We used a weighted Whittaker smoothing process [75,76] to remove inherent noise in NDVI data [77]. All NDVI interpolations, standardizations, and smoothing processes were conducted in R [78].

### 2.6. Statistical Analyses

We found temporal differences in adult female pronghorn nutrition and diet among these subpopulations [31,63]; therefore, we analyzed our data separately for each sampling period to examine potential differences in predictor variables on fawn recruitment across adult female life history stages of pronghorn in Idaho. Our analyses involved pronghorn fawn recruitment estimates as the response variable and 18 predictor variables (Table 2). Predictor variables included the aforementioned intrinsic and extrinsic factors, as well as their standard deviations, and were averaged by subpopulation and year for analyses. Fawn recruitment estimates were recorded as ratios where we divided the total number of fawns by the total number of adult females observed per subpopulation (Table 1). Variation and predictability of environmental factors and plant phenology were found to be important in ungulate demographics [79,80]; therefore, we included the standard deviation (SD) of our parameters as a measure of variability across individuals within a subpopulation for each measure.

We first assessed correlations via Pearson’s correlation tests, using the cor.test command, to evaluate within-year relationships between predictor variables from late gestation and early lactation and fawn recruitment, as well as predictor variables from the breeding season and the following year’s recruitment. Breeding season sampling occurred after recruitment estimates were determined (i.e., lag effect). Our first step involved eliminating variables by carrying forward only those with correlation coefficients > 0.50 for regression analyses in R. We then used a model selection approach to determine the influence of those predictor variables, by sampling period, on pronghorn fawn recruitment in Idaho. Dietary variables (i.e., protein intake of forb, graminoid, legume, shrub) were recorded as proportions (i.e., 0.0–1.0 scale) where each sample summed to 1 across plant functional groups. Variables with a correlation coefficient > 0.50 were not allowed to occur in the same multivariate model [81]. We ran those selected variables in univariate and additive regression models, along with the null model, to assess whether the inclusion of additive models increased explanatory power for each sampling period. Our null model was fawn recruitment as the response variable. The equation of the null model was lm(formula = recruitment~1).

We used Akaike’s information criterion for small sample sizes (AICc) and AICc weights to rank models. We evaluated the AICc weights of the top-ranking model(s) and the null model to describe the strength of evidence for the top model(s) [82]. We did not combine variable means and variances in additive models, given their inherent correlation. We considered models equivalent when the ratio between AICc weights was <2.0, which would indicate that the top-ranking model was less than twice as likely as the null [83]. We tested models for normality of residuals, heteroscedasticity of residuals, and equal variance. We used α = 0.10 for all statistical tests. With the amount of variability in most study systems and the general lack of controls or replications, statistical tests producing *p*-values of <0.10 should be examined thoroughly as a biological relationship may exist among the parameters measured, and sample size may be limiting any definitive statistical conclusion [84].

## 3. Results

We removed two samples from our analyses due to contamination issues with plant DNA barcoding analyses, resulting in a sample size of 558 for this study. Estimates of fawn recruitment varied by subpopulation and year (Table 1). We found temporal differences in the number of predictor variables per sampling period at six, two, and four for late gestation, early lactation, and breeding season, respectively. Herd composition survey results for totals and age ratios from each year can be found in Table 3.

### 3.1. Late Gestation

**Figure 2:**
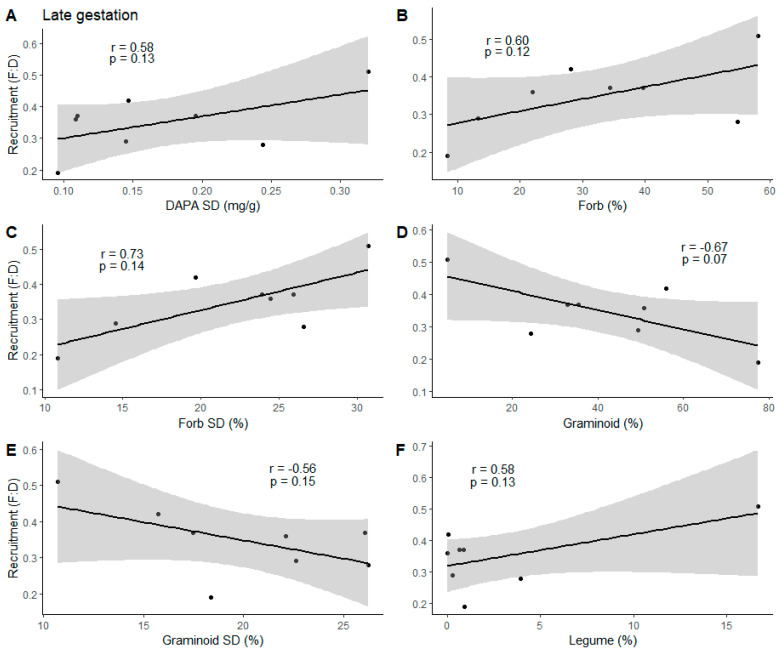
Correlations between (**A**) standard deviation of 2,6-diaminopimelic acid (DAPA SD (mg/g)), (**B**) mean forb protein intake (Forb (%)), (**C**) standard deviation of forb protein intake (Forb SD (%)), (**D**) graminoid protein intake (Graminoid (%)), (**E**) SD of graminoid protein intake (Graminoid SD (%)), and (**F**) legume protein intake (Legume (%)) values, and pronghorn fawn recruitment (i.e., fawn:doe ratio (F:D)) estimates, from late gestation sampling across five subpopulations in Idaho, 2018–2019. The black line represents the regression line, and the gray shaded area is the 95% confidence interval. Black dots (*n* = 8) represent a mean value for each subpopulation for each year.

The standard deviation of DAPA (DAPA SD), dietary protein intake of forbs (Forb), the standard deviation of forb protein intake (Forb SD), dietary protein intake of graminoids (Graminoid), the standard deviation of graminoid protein intake (Graminoid SD), and dietary protein intake of legumes (Legume) variables were carried forward for late gestation. From those six variables, we found pronghorn fawn recruitment to be negatively correlated with protein intake of graminoids (r = −0.67, df = 6, *p* = 0.07; Figure 2) during late gestation. We detected significant correlations between DAPA SD and Forb (r = 0.87, *N* = 8, *p* = 0.02), DAPA SD and Legume (r = 0.86, *N* = 8, *p* = 0.006), Legume and Forb (r = 0.69, *N* = 8, *p* = 0.05), and DAPA SD and Forb SD (r = 0.70, *N* = 8, *p* = 0.05) variables during late gestation analyses and therefore Legume and Forb SD was the only additive model. Forb SD (R^2^ = 0.53, F_1, 6_ = 6.90, *p* = 0.04) was the top model during late gestation containing 40% of the model weight (Table 4). The null model had a ΔAICc of 0.52.

### 3.2. Early Lactation

**Figure 3:**
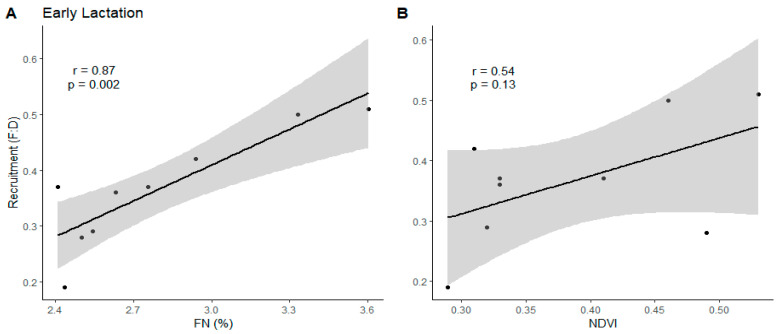
Correlations between (**A**) mean fecal nitrogen (FN (%)) and (**B**) normalized difference vegetation index (NDVI) values, with pronghorn fawn recruitment (i.e., fawn:doe ratio (F:D)) estimates from early lactation sampling across five subpopulations in Idaho, 2018–2019. The black line represents the regression line, and the gray shaded area is the 95% confidence interval. Black dots (*n* = 9) represent a mean value for each subpopulation for each year.

Fecal nitrogen was significantly correlated (r = 0.87, df = 7, *p* = 0.002) with pronghorn fawn recruitment during early lactation (Figure 3). We also carried forward NDVI for regression analysis based on the strength of the relationship with fawn recruitment (r = 0.54, df = 7, *p* = 0.13). Fecal nitrogen and NDVI were correlated (r = 0.56, df = 7, *p* = 0.12) and not included in regression analysis. Fecal nitrogen was the best model (R^2^ = 0.76, F_2, 6_ = 9.46, *p* = 0.01) in predicting pronghorn fawn recruitment in all study subpopulations and contained 95% of the model weight (Table 4). All remaining models had ΔAICc > 2.0.

### 3.3. Breeding Season Lag Effect

**Figure 4:**
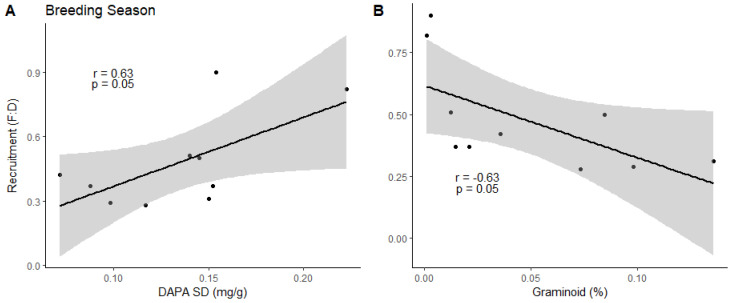

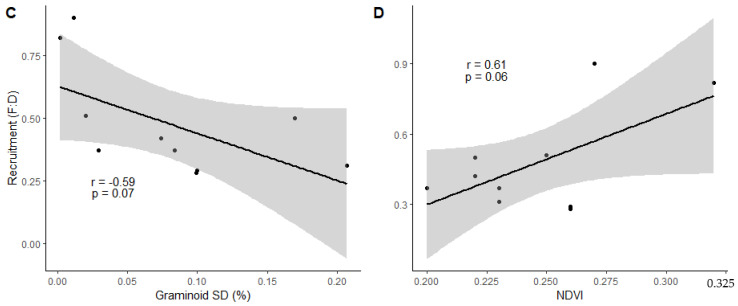
Correlations between (**A**) standard deviation of 2,6-diaminopimelic acid (DAPA SD (mg/g)), (**B**) graminoid protein intake (Graminoid (%)), (**C**) SD of graminoid protein intake (Graminoid SD (%)), and (**D**) normalized difference vegetation index (NDVI) values from the previous breeding season, with the succeeding year’s pronghorn fawn recruitment (i.e., fawn:doe ratio (F:D); 2019–2020) estimates across five subpopulations in Idaho, 2018–2019. The black line represents the regression line, and the gray shaded area is the 95% confidence interval. Black dots (*n* = 9) represent a mean value for each subpopulation for each year.

Graminoid (r = −0.63, df = 8, *p* = 0.05) and Graminoid SD (r = −0.59, df = 8, *p* = 0.07) during breeding season were negatively correlated with the following year’s fawn recruitment while DAPA SD (r = 0.63, df = 8, *p* = 0.05) and NDVI (r = 0.61, df = 8, *p* = 0.06) were positively correlated (Figure 4). DAPA SD and NDVI variables were carried forward for breeding season lag effect regression analysis, but this additive model was correlated (r = 0.70, *N* = 10, *p* = 0.02) and not included. DAPA SD was the top model (R^2^ = 0.40, F_1, 8_ = 5.37, *p* = 0.05) for breeding season effect on the following year’s fawn recruitment containing 41% of the model weight, but the NDVI and null models had ΔAICc < 2.0 (Table 4). Additive models demonstrated that the addition of variables did not improve the explanatory power of any models.

## 4. Discussion

### 4.1. Late Gestation

Fawn recruitment was positively associated with late gestation protein intake of forbs and legumes as well as the variability in forb protein intake and DAPA (Figure 2A–C,F), but negatively related to protein intake of graminoids and the variability in graminoid protein intake (Figure 2D,E). The relationships between diet variables and fawn recruitment may have reflected plant functional group availability, but no information is available in this regard. The negative relationship with graminoids is likely a result of the animals’ small rumen and associated inability to digest plants with high content of lignocellulose and fiber (i.e., graminoids; [53]). Birch Creek and Jarbidge subpopulations consumed the highest proportions of protein intake from graminoids and subsequently had lower recruitment estimates compared to the Camas Prairie subpopulation, where a majority of protein intake from this subpopulation was from forbs and showed higher recruitment estimates. The Camas Prairie subpopulation was the only site to obtain >4.0% of dietary protein from legumes during late gestation, with a mean composition of 16.67% of plant species consumed, primarily driven by sainfoin (*Onobrychis viciifolia*; [65]). Forbs are high in crude protein content [85] and have been described as “production plants” because years of higher fawn recruitment coincide with high abundance and extenuated succulence of forbs throughout the summer [86]. Such increased proportion and higher recruitment estimates likely influenced the relationship between legume protein intake and fawn recruitment during this time. Pronghorn diet studies are generally typified into major plant functional groups (i.e., graminoids, forbs, shrubs; [86]), but recent work included legumes as a separate group, which enabled the investigation of potential spatially explicit impacts on agricultural lands. Bleke et al. [62] utilized plant DNA barcoding for dietary analysis and found pronghorn within the shrub–steppe biome acquired the highest proportion of protein from graminoids and the lowest from forbs during late gestation.

Our late gestation sample collection occurred during the third trimester of gestation, the most metabolically demanding period of pregnancy [37], when metabolic costs are 50% greater in pregnant females than in nonpregnant females [87]. Pronghorn pregnant with twins are believed to invest more in reproduction than all other ungulate species per reproductive event [88,89]. Pregnant pronghorn require highly digestible, high-protein forbs during this time as 50% of fetal mass development occurs during the final month of gestation [50], constraining the amount of biomass that could be ingested and digested on a daily basis [90]. In turn, the female’s nutritional status directly influences neonate weight and subsequent survival rates [38,91,92]. 

### 4.2. Early Lactation

Fawn recruitment was positively related to early lactation fecal nitrogen and NDVI (Figure 3A,B). Fecal nitrogen was the top model during early lactation, with 97% of the model weight, meaning as subpopulation fecal nitrogen increased, fawn recruitment increased (Table 4). Fecal nitrogen and fecal crude protein share a linear relationship [93] such that as diet quality (i.e., fecal nitrogen) increases, so does protein intake. The importance of fecal nitrogen during this time is likely a result of adult female metabolic demands during early lactation.

The transition from gestation to lactation is among the most important determinants of maternal–offspring health outcomes [94,95,96], when daily energetic requirements increase by 65–215% during the first month postpartum [97,98], and is the metabolic peak for female ungulates [9]. Peak milk volume and milk energy output, in proportion to maternal weight, are higher in pronghorn than other studied species of North American big game [99], particularly during the first two weeks of lactation [86].

Growth rates of neonatal pronghorn have been directly related to milk energy intake, which is highest during early lactation, and a direct result of adult female body condition [100]. The rapid growth rates of pronghorn fawns enable them to quickly attain adult proportions, improving their locomotion efficiency and subsequently increasing survival by outrunning predators [100]. This directly supports our findings on the critical importance of diet quality to female pronghorn during lactation and its direct influence on fawn survival and recruitment. The response of fawn growth rates to a range of energy and protein intake levels suggests nutrition, rather than physiology, constrains the rate of fawn development [99]. Poor forage quality may reduce female milk production, thereby reducing fawn growth rates and subsequently increasing the period of time in which fawns are vulnerable to predation [47].

Fecal nitrogen was highest during early lactation [31], which is supported by previous nutrition work conducted amongst these subpopulations [47]. Both studies found the Camas Prairie subpopulation to have the highest fecal nitrogen during this time, while Jarbidge or Little Wood subpopulations had the lowest values, depending on the year of sampling [31,47]. Forb protein intake [62] and fawn recruitment estimates shared a similar pattern, indicating that increasing diet quality, or forbs, available to pronghorn may be a management strategy to increase fawn recruitment. The annual metabolic commitment that female pronghorn make to fawn recruitment and the influence of dietary nutritional quality on female condition [68] stresses the value of using adult female fecal nitrogen as a predictor of recruitment.

### 4.3. Breeding Season Lag Effect

Breeding season variations in DAPA and NDVI were positively related to the following year’s fawn recruitment, while protein intake of graminoids and its variation were negatively related (Figure 4). This negative relationship is likely the result of constraints that a small rumen imposes on fiber digestion, as previously explained [53]. Subpopulation variation in DAPA contained 41% of the model weight, but NDVI and the null model had ΔAICc < 2.0 (Table 4), preventing us from deeming it the top model. DAPA variation was higher in 2019 compared to 2018, and fawn recruitment estimates were higher for most subpopulations in 2019, which may help in explaining why DAPA SD was the top model. Variations in DAPA and NDVI were correlated during this period as well and this positive association is likely complementary and aids in female pronghorn’s ability to metabolically recover from the costs of reproduction and lactation [101,102] when female milk energy output is greatly reduced [86].

Female pronghorn generally exhibit no indication of reduced reproductive success attributable to the previous reproductive event(s) [101], yet they produce twins each year [102] at a time when plant quality has declined due to maturation and senescence [103]. This is not the case with other ungulates, such as bighorn sheep (*Ovis canadensis*), where reproductive success in one year leads to lower condition and decreased success the following year [104]. Female ungulates are generally in their best condition at the onset of winter when metabolic demands have greatly decreased following the weaning of young [37]. Specifically, pronghorn maternal condition during fall estrus has been shown to influence offspring prenatal growth rates [102], gestation length, and birth weight [105], further supporting the importance of digestible energy for female pronghorn during the breeding season.

While we demonstrated the utility of a noninvasive technique to predict summer fawn survival for pronghorn in northern latitudes, it did not address the possible effects of predation, habitat quality, or weather. Panting et al. [92] investigated pronghorn neonate survival in Idaho and found neonate body mass index, lagomorph abundance, and diet quality during gestation and lactation influence fawn survival. Our results support this finding. Smyser [47] postulated that summer range quality is a limiting factor for these subpopulations. Our use of DNA barcoding for diet determination allowed us to determine temporal plant functional group protein intake and its influence on fawn summer survival. Advances in the GenBank software will increase the taxonomic resolution of plants consumed and highlight the temporal and spatial importance of particular plant species. Gese et al. [68] explored environmental influence on pronghorn fawn recruitment in Idaho and found that the climatic drivers were spatially and temporarily explicit to the elevation each subpopulation occupied. Finally, our small sample size limited the statistical inferences that could be made on correlation results. Our interest was in modeling variables that showed moderate relationships with recruitment to then determine which had the strongest influence.

## 5. Conclusions

To noninvasively monitor pronghorn fawn recruitment in Idaho, we recommend managers collect fecal samples during the early lactation period (i.e., June) to assess baseline fecal nitrogen content. Fresh fecal samples are required for accurate hormone measurements due to degradations via exposure [63], but retention of nitrogen in feces is not compromised by exposure to weather and insects for 2–3 weeks post-defecation [106], making fresh collections unnecessary but highly encouraged. Even with a small sample size, fecal nitrogen accounted for 97% of the model weight for all subpopulations and years combined. While June corresponded with early lactation in northern pronghorn populations, it is likely that the life-history stage is more important than the calendar month. Broadly, pronghorn fawn recruitment is a function of fawn growth rates, which depends on fawn milk energy intake. This variable is, in turn, a function of female milk energy output, which relies on diet quality and plant productivity. As such, management decisions should be made at the subpopulation level, where actions should be aimed at increasing diet quality, particularly forbs available during early lactation, for those subpopulations with low fawn recruitment. We showed that any increase in nitrogen on the landscape for lactating adult females may positively influence fawn recruitment.

Temporal variation in the relationships between pronghorn recruitment and physiological variables showed differences in the seasonal demands of adult females. Given our sampling design, we were able to examine the relationships of our measured variables and known adult female expenditures: (a) variation in digestible energy and plant productivity on maternal condition during estrus; (b) variation in digestible energy and diet composition on prenatal growth rates and birth weights during late gestation; and finally, (c) diet quality on milk energy output and associated fawn growth rates and subsequent fawn recruitment during early lactation. Sampling designs utilizing multiple concurrent life-history stages of adult females enable managers to longitudinally track and monitor drivers or factors influencing vital rates (e.g., fawn recruitment) within populations of interest. Improving our understanding of seasonal patterns or differences in ungulate nutritional conditions and their effects on population dynamics can provide useful insight for and guide population and habitat management [37,107,108,109]. Pronghorn in this study consumed plants from all functional groups (i.e., forbs, graminoids, legumes, shrubs) with proportions varying across female life history stages [62], demonstrating the importance of vegetative communities. That balance is important given the differing relationships between protein intake of plant functional groups and fawn recruitment.

Noninvasive monitoring of wildlife populations is increasing in use and popularity, given the feasibility and insight it provides without requiring the capture of an animal. We believe our methodologies and results presented within this study can be broadly tested and applied to ungulate monitoring programs, particularly for elusive and endangered species or species occupying habitats difficult to survey. Species such as pronghorn are at increased risk of injury during capture events, justifying the need for continued adoption of these techniques.

## Figures and Tables

**Table 1:** Mean elevation, annual precipitation, monthly maximum and minimum temperatures, growing degree days (GDDs), and fawn recruitment (fawn:doe) estimates of summer ranges of pronghorn subpopulations in Idaho, 2018–2020.

Subpopulation	Mean Elevation (m)	Mean Annual Precipitation (cm)	Mean Monthly Max Temperature (°C)	Mean Monthly Min Temperature (°C)	GDDs (2018)	GDDs (2019)	2018 Recruitment	2019 Recruitment	2020 Recruitment
Birch Creek	2018	23.72	27.78	−15.56	1967	1851	0.36	0.42	0.5
Camas Prairie	1552	33.66	29.44	14.44	1893	2005	0.5	0.51	0.82
Jarbidge	1552	24.41	31.67	−6.11	1789	1457	0.19	0.37	0.37
Little Wood	1726	32.89	29.44	−13.33	2385	2218	-	0.28	0.9
Pahsimeroi	1897	19.76	31.11	−14.44	2579	2349	0.37	0.29	0.31

**Table 2:** Intrinsic and extrinsic predictor variables and their variation (SD), hypothesized to influence pronghorn fawn summer survival in southern Idaho, USA, 2018–2020. Predictor variables were associated with concurrent late gestation and early lactation sampling periods as well as the following year’s breeding season. The Other functional group contained the families Orobanchaceae, Rosaceae, and Asteraceae, with each comprising forbs and shrubs, which prevented us from classifying them into a specific functional group (i.e., forbs, graminoids, legumes, shrubs).

Type	Variable ^1^	Description
Intrinsic	FN	Mean FN of samples
Intrinsic	FN SD	Standard deviation of FN of samples
Intrinsic	DAPA	Mean DAPA of samples
Intrinsic	DAPA SD	Standard deviation of DAPA of samples
Intrinsic	FGM	Mean FGM of samples
Intrinsic	FGM SD	Standard deviation of FGM of samples
Intrinsic	Forb	Mean proportion of dietary protein intake from forbs of samples
Intrinsic	Forb SD	Standard deviation dietary protein intake from forbs
Intrinsic	Graminoid	Mean proportion of dietary protein intake from graminoids of samples
Intrinsic	Graminoid SD	Standard deviation dietary protein intake from graminoids
Intrinsic	Legume	Mean proportion of dietary protein intake from legumes of samples
Intrinsic	Legume SD	Standard deviation dietary protein intake from legumes
Intrinsic	Shrub	Mean proportion of dietary protein intake from shrubs of samples
Intrinsic	Shrub SD	Standard deviation dietary protein intake from shrubs
Intrinsic	Other	Mean proportion of dietary protein intake from other functional group of samples
Intrinsic	Other SD	Standard deviation dietary protein intake from other functional group
Extrinsic	NDVI	Temporal mean NDVI of subpopulation summer ranges
Extrinsic	NDVI SD	Spatial variation in NDVI of subpopulation summer ranges

^1^ FN = fecal nitrogen; DAPA = 2,6-diaminopimelic acid; FGM = fecal glucocorticoid metabolite; NDVI = normalized difference vegetation index.

**Table 3:** Results of pronghorn herd composition surveys in southern Idaho, USA, 2018–2020. Each year included totals of fawns and adult females as well as calculated fawn:doe ratios.

	Birch Creek			Camas Prairie			Jarbidge			Little Wood			Pahsimeroi		
Year	2018	2019	2020	2018	2019	2020	2018	2019	2020	2018	2019	2020	2018	2019	2020
Fawns	42	126	122	82	90	126	38	72	80	-	18	38	69	135	126
Adult females	118	216	246	165	175	153	203	193	216	-	64	42	187	466	153
Young:Adult female	0.36	0.58	0.5	0.5	0.51	0.82	0.19	0.37	0.37	-	0.28	0.90	0.37	0.29	0.82

**Table 4:** Models for linear regression analyses of predictor variables on pronghorn fawn recruitment by sampling period (late gestation, early lactation, breeding season) in southern Idaho, 2018–2019. We present the number of parameters (K), Akaike’s information criterion corrected for small sample sizes (AICc), differences in AICc (ΔAICc), and model weight (wi).

Model ^1^	K	AIC_c_	ΔAIC_c_	w_i_	R^2^
Late gestation model					
Forb SD	3	−9.86	0	0.40	0.53
Null	2	−9.34	0.52	0.30	
Forb	3	−7.27	2.59	0.11	0.36
Legume	3	−7.22	2.64	0.11	0.33
DAPA SD	3	−6.68	3.18	0.08	0.31
Legume + Forb SD	4	−1.30	8.56	0.01	0.57
Early lactation model					
FN	3	−18.19	0	0.97	0.76
Null	2	−10.33	7.86	0.02	
NDVI	3	−8.63	9.57	0.01	0.29
Breeding season model					
DAPA SD	3	1.69	0.00	0.41	0.28
NDVI	3	2.22	0.54	0.32	0.34
Null	2	2.53	0.85	0.27	

^1^ Covariates include standard deviation of 2,6-diaminopimelic acid (DAPA SD), fecal nitrogen (FN), forb protein intake (Forb), standard deviation of forb protein intake (Forb SD), legume protein intake (Legume), and normalized difference vegetation index (NDVI).

## Data Availability

The dataset in this study can be requested from the corresponding author.
